# Disease Pandemics and Major Epidemics Arising From New Encounters between Indigenous Viruses and Introduced Crops

**DOI:** 10.3390/v12121388

**Published:** 2020-12-04

**Authors:** Roger A. C. Jones

**Affiliations:** Institute of Agriculture, University of Western Australia, 35 Stirling Highway, Crawley, WA 6009, Australia; roger.jones@uwa.edu.au

**Keywords:** pandemics, epidemics, global, disease, threat, food insecurity, crop losses, crop failure, indigenous viruses, introduced crops, new encounter, spillover, developing countries, domestication centers, sub–Saharan Africa

## Abstract

Virus disease pandemics and epidemics that occur in the world’s staple food crops pose a major threat to global food security, especially in developing countries with tropical or subtropical climates. Moreover, this threat is escalating rapidly due to increasing difficulties in controlling virus diseases as climate change accelerates and the need to feed the burgeoning global population escalates. One of the main causes of these pandemics and epidemics is the introduction to a new continent of food crops domesticated elsewhere, and their subsequent invasion by damaging virus diseases they never encountered before. This review focusses on providing historical and up-to-date information about pandemics and major epidemics initiated by spillover of indigenous viruses from infected alternative hosts into introduced crops. This spillover requires new encounters at the managed and natural vegetation interface. The principal virus disease pandemic examples described are two (cassava mosaic, cassava brown streak) that threaten food security in sub-Saharan Africa (SSA), and one (tomato yellow leaf curl) doing so globally. A further example describes a virus disease pandemic threatening a major plantation crop producing a vital food export for West Africa (cacao swollen shoot). Also described are two examples of major virus disease epidemics that threaten SSA’s food security (rice yellow mottle, groundnut rosette). In addition, brief accounts are provided of two major maize virus disease epidemics (maize streak in SSA, maize rough dwarf in Mediterranean and Middle Eastern regions), a major rice disease epidemic (rice hoja blanca in the Americas), and damaging tomato tospovirus and begomovirus disease epidemics of tomato that impair food security in different world regions. For each pandemic or major epidemic, the factors involved in driving its initial emergence, and its subsequent increase in importance and geographical distribution, are explained. Finally, clarification is provided over what needs to be done globally to achieve effective management of severe virus disease pandemics and epidemics initiated by spillover events.

## 1. Introduction

Virus disease epidemics and pandemics threaten all types of cultivated plants including those grown to feed the world’s human population and its domestic animals, and others grown for ornamental, fiber or medicinal uses [1,2,3,4,5,6,7]. Virus epidemics also threaten wild plant communities growing in natural ecosystems [8,9,10,11,12,13]. With crop plants, they diminish the growth and vigor of infected plants, decrease gross yields and disfigure plant produce. The losses they cause vary from total crop failure to smaller scale, occur worldwide and have an estimated economic global impact of >US$30 billion annually [1,2,6,7,14,15,16,17,18]. They occur in all types of crop plants. These include staple food crops of crucial significance for achieving food security in subtropical and tropical regions [1,4,5,7,19,20,21,22,23,24,25]. With mixed species-managed pastures and wild plant communities in natural ecosystems, their detrimental effects on the growth and vigor of infected plants alter plant species composition. In managed pastures, they diminish the proportion of pasture plants versus weeds causing pasture deterioration and an inadequate feed base for livestock [26,27,28,29,30,31,32,33]. In wild plant communities, they alter the species balance and decrease species diversity, which damages ecosystems and can cause genetic erosion potentially leading to species extinction [12,13,34,35,36,37].

Development of damaging virus epidemics is favored by the introduction of new crops to parts of the world where they have never been grown before and the adoption of intensive cropping systems both of which lead to new encounters with virulent viruses infecting crops or indigenous vegetation. They are also favored by introduction of vulnerable new cultivars bred for increased yields [1,2,4,19,20,38,39,40]. In mixed species-managed pastures, damaging virus epidemics are favored by factors such as relative grazing pressure and trampling by domestic animals resulting in increased insect vector numbers and virus spread by vectors or contact transmission [31,32,33]. In wild plant communities, they are aggravated by factors such as fragmentation into small patches of vegetation enclosed by crops or urban areas, livestock grazing and human disturbance, e.g., woodcutting and flower collection [4,9,10,37,41].

Several of the world’s plant virus disease pandemics and major epidemics have resulted from infection with emerging viruses that arose from new encounter situations in which indigenous viruses spread by spillover (= host species jumps) from infected indigenous plants to infect introduced cultivated plants [1,4,5,7,19,20,42]. However, epidemics can also take place when introduced viruses spread to indigenous plants from infected introduced cultivated plants [4,9,10,11,12,13,37]. Thus, on the one hand, when introduced cultivated plants domesticated elsewhere grow next to indigenous wild plants or locally domesticated crop plants they never encountered previously, indigenous viruses associated with these indigenous hosts can spillover to the introduced crop plants causing virus disease epidemics in them. On the other hand, introduced viruses can also spread to indigenous crop or wild plants from infected introduced cultivated plants or associated weeds, causing virus epidemics. Both types of invasions require virus spread to occur at the interface between indigenous and introduced plants [1,4,9,10,11,12,13,37,40,41,42,43,44].

Pandemics or epidemics occurring in diverse crops and all continents, apart from Antarctica, were documented in a series of reviews written by the late Professor Michael Thresh [45]. These reviews covered the period from the inception of plant virology in the early 1900s up to 2006 [1,2,19,20,38,40,46,47,48,49,50]. In 1980, Thresh [1] provided a review of the origins and epidemiology of a wide range of important plant virus diseases. More up-to-date accounts of damaging pandemics or major epidemics involving several mostly single virus–host–vector pathosystems were described in several recent reviews [51,52,53,54,55,56,57,58]. In addition, a recent review focused on the global dimensions of plant virus disease [7]. 

This review describes virus disease pandemics and major epidemics that arose from spillover scenarios involving new encounters between indigenous viruses and introduced crops, rather than virus spread from introduced crops to indigenous crops or natural vegetation. It does this by providing historical and up-to-date information on five examples of virus diseases that threaten staple food crops critically important for food security in developing countries, placing special emphasis on the situation in sub-Saharan Africa (SSA). The sixth virus disease example threatens livelihoods in SSA because it devastates production of a valuable food export crop. In addition, brief coverage is provided of several other examples of major virus disease epidemics that arose from new encounters between indigenous viruses and introduced crops important for food security in different parts of the world.

## 2. General Concepts 

### 2.1. Definitions 

In his 1970 review of ‘catastrophic plant diseases’, Klinkowski [59] emphasized that many plant disease agents, including viruses, cause epidemics and pandemics, especially when they spread from their centers of origin into continents where they were formerly absent. He defined an epidemic as being “where a disease is spread over an area in which its causal agent has been present for a long time”; a progressive epidemic as “where it expands from this area into others”; and a pandemic as “where epidemics cause mass infections spread over several continents”. He gave five plant virus disease examples: sugarcane mosaic disease spreading worldwide fitted his ‘pandemic’ definition; plum pox, sugar beet yellows and tobacco veinal necrosis diseases spreading mostly in Europe matched his progressive epidemic definition; and cocoa swollen shoot disease (CSSD) spreading in Ghana, West Africa matched his epidemic definition. Subsequently, in plant virology, the term progressive epidemic has fallen into disuse and a plant virus disease pandemic has come to include “an epidemic occurring over a very wide area, crossing international boundaries and causing severe crop losses” [23]. In practice, however, the term epidemic is now widely used to cover all three of these types of epidemic situations, while the term pandemic has become restricted mainly to damaging virus diseases that spread widely between different countries in SSA, e.g., CSSD [18] and cassava mosaic disease (CMD) [23] and cassava brown streak disease (CBSD) [52]. In this review, the ‘pandemic’ definition now mainly used in Africa is also applied to other continents, otherwise the term ‘epidemic’ is used. 

An emerging virus is usually considered to be “one that causes damaging epidemics but has only evolved or been recognized recently, changed its pathogenesis, increased its host range or increased its geographical distribution” [3,55]. Further, a re-emerging virus is usually considered to be “one that once caused serious disease problems, but then declined in importance before suddenly increasing in incidence and geographical distribution causing considerable crop damage” [4]. Therefore, the term virus emergence refers to “the first appearance of a virus and its associated initial increase in incidence/geographic range”, and the term virus re-emergence refers to “the reappearance of virus and its associated increase in incidence/geographic range”. When the term vulnerable is applied to a crop cultivar [20], this means that “the cultivar is both susceptible to virus infection (i.e., it becomes infected readily), and sensitive to infection once systemic infection has occurred (i.e., it develops severe symptoms)” [1,60]. Thus, susceptible is the opposite of resistance and sensitive is the opposite of tolerance [60]. The term virus spillover refers to “spread of a virus from naturally-infected host to a new host it has not encountered previously”, and the term spillback refers to “spread of a virus from the new host back to the natural host” [42]. 

### 2.2. Crop Domestication Centers and Introductions

Selection of local land races of crop plants from wild ancestors commenced more than 10,000 years ago in the worlds’ plant domestication centers [61,62]. Viruses from these wild ancestors were present among the land races derived from them and these indigenous viruses adapted to their new situation multiplying in cultivated plants growing mostly in mixed species cultivation [1,4,9]. Later, through international trade, crop plants were moved progressively away from their domestication centers to distant continents where they were often grown as monocultures. For example, the Columbian Exchange was responsible for the introduction of crops critical for food security to other continents following the Spanish 1492 arrival in the Americas, such as maize (*Zea mays*), cassava (*Manihot esculenta*), potato (*Solanum tuberosum*) and tomato (*Solanum lycopersicum*) [63]. In consequence, new encounters between introduced cultivated plants, and infected wild or crop plants occurred resulting in spillover of indigenous viruses into introduced crops. Sometimes epidemics arose soon afterwards and sometimes only after a considerable delay triggered by other factors, and some later became pandemics [1,4,9,18,19,20,21,22,23,40,52]. 

### 2.3. Factors Favoring Spillover

Successful spillover starts with spread of already existing genetic virus variants from a virus infection source plant to the new host plant, and the outcome for each individual variant depends on its relative abilities (i.e., fitness) to survive once it infects each host, adapt to new hosts or vectors and achieve efficient epidemic spread [64]. A range of factors favor successful virus spillover, emergence or re-emergence. These include: presence of efficient indigenous or introduced virus vectors, including “supervectors”; introduction of vulnerable crop cultivars; adoption of cultural practices involving agricultural intensification, extensification and diversification; intensive wildflower production and conservation projects; the relative ability of a virus to generate virulent new variants through mutation, reassortment and recombination; and climate change arising from global warming [1,2,4,16,19,20,42,55,64,65,66,67,68,69,70,71,72,73].

## 3. Rice Yellow Mottle Disease 

Asian rice (*Oryza sativa*) is a cereal crop domesticated from wild rice in China approximately 10,000 years ago. It soon spread from there to Southeast Asia, the rest of East Asia and the Indian subcontinent, next to the Middle East, Europe and North Africa, and more recently to the Americas and Oceania. Approximately 1000 years ago, it was introduced to East Africa where it was grown in coastal regions. In the second half of the 19th century, it was taken inland to be sown in the rest of East Africa, Central Africa and then taken to West Africa and Madagascar. The inland delta of the upper Niger River was where African rice (*Oryza glaberrima*) was first domesticated 3000 years ago. It spread gradually from there within West Africa [74,75]. Overall, rice is ranked as third in importance as a staple food crop but in the developing world it is ranked first [76,77]. Many viruses cause disease epidemics in this crop [78]. An example of a major rice virus disease epidemic that arose by virus spillover and now endangers developing country food security is described below.

Rice yellow mottle disease (RYMD) was first described in 1966 infecting rice plantings in the Lake Victoria region of Kenya in East Africa (Table 1). This initial appearance coincided with one of Africa’s first intensive irrigated rice production programs. Afterwards, on several different occasions, such programs triggered RYMD appearance in other locations in both East and West Africa. RYMD then spread to most rice-growing countries in other parts of SSA and by 1989 had spread to the island of Madagascar [1,55,75,79,80]. Since the mid-1990s, it has caused a disease epidemic of major economic significance in rice-growing regions and become a major deterrent to rice cultivation in SSA. Both irrigated and rainfed rice develop RYMD but its incidences are higher in irrigated crops [1,55,75,78,79,80,81]. However, it has not yet spread elsewhere in the world. RYMD foliage symptoms in rice consist of leaf yellowing, plant stunting, diminished tillering and poor panicle filling, and are associated with low seed production and poor grain quality. The disease causes yield losses of 25–100% [55,78].

The causal agent of RYMD is rice yellow mottle virus (RYMV; genus, *Sobemovirus*, family, *Sobemoviridae*). RYMV infection occurs naturally in cultivated African and Asian rice, the wild rice species *O. barthii* and *O. longistaminata*, and the wild grasses *Echinocloa colona*, *Eragrostis atrovirens* and *Panicum repens* [78,79]. RYMV has stable spherical virions that remain infectious for long periods on contaminated surfaces and reach high concentrations in infected plants [78]. It is therefore readily contact transmitted, including by wind-meditated plant-to-plant contact transmission [55,100]. It is also transmitted by several chrysomelid beetle species, its most efficient beetle vector being *Sesselia pussilla*. In addition, it is transmitted by mammals and in irrigation water and soil, but is not seed transmitted to seedlings [1,55,75,78,79,100]. Carry over between cropping periods occurs mainly in infected rice stubble arising from incompletely decomposed contaminated plant debris, allowing crops to regenerate from tillers growing from these stubbles (ratooning) and infected wild hosts [75,78,79]. 

Up until the 1960s, rice was only grown in small-scale subsistence plantings in SSA. In the 19th century in coastal East Africa, RYMV emerged in Asian rice plantings via virus spillover from nearby wild rice and grass hosts, and then spread inland. In West Africa, at the end of the 19th century, a similar spillover process resulted in its emergence in African rice plantings in the upper Niger River delta region, and its spread elsewhere in this region. In both instances, its emergence was attributed to its spread by contact and vectors to rice, and intensification of rice production at the natural and managed vegetation interface under subsistence farming conditions [75]. The introduction of large-sale, intensive irrigated rice production schemes, including irrigation over much of SSA led to its initial detection in Kenya in 1966, development of a major RYMD epidemic and the resulting widespread severe production losses in most rice-growing SSA countries. Irrigation allowed extensive growth of volunteer cultivated rice, wild rice and weed grass plants that remained present during the dry season providing an infection reservoir for RYMV spread in the following growing season [1,75,79]. 

What was responsible for the increase in geographical distribution of RYMV infection in rice crops found since 1966 within SSA? Since RYMV is not seed borne, widespread dissemination via the seed trade can be discounted. Although spread by vectors from infected alternative hosts or via contaminated irrigation water, soil containing plant debris or agricultural machinery could account for local spread, but they would not account for its rapid long-distance dissemination. Rakotomalala et al. [81] suggested that the rice trade might have been responsible for spreading RYMV from continental Africa to Madagascar. Thus, unknowingly transporting RYMV-infected live rice seedlings, stubble or ratoons to Madagascar, and planting them there, would have introduced the virus. Such introduction via trade could also explain its spread from one country to another within continental Africa, but direct evidence of what actually occurred is lacking [75,79]. Since rice is ranked as the most important staple food crop in the developing world (see above), spread of RYMV to other rice-growing regions of the world leading to a major global epidemic would constitute a further cause for concern over future food security.

## 4. Cassava Mosaic Disease

Cassava is a perennial tuberous root crop domesticated 10,000 years ago in the Amazonian rain forest region of South America. It is ranked fifth in global importance as a staple food crop, and is currently the third most important food staple in developing countries [77,101] where it is mainly grown by smallholder farmers [54]. In the 16th century, it was taken to West Africa. By the beginning of the 19th century, it was being grown throughout West, Central and East Africa, and had also been introduced to the Indian subcontinent and Southeast Asia. During the 20th century, its cultivation greatly increased in SSA and southern Asia. Africa is now responsible for more than half of its global production. It is propagated vegetatively and grows well in the world’s tropical regions, tolerates poor soils and drought, requires minimal inputs, and delivers a high output of energy per hectare [77,101]. Cassava crops become infected with several virus diseases [82]. Two examples of devastating cassava virus disease pandemics that arose by virus spillover and are now endangering food security in developing countries are described below in this section, and in Section 5. 

**Figure 1:**
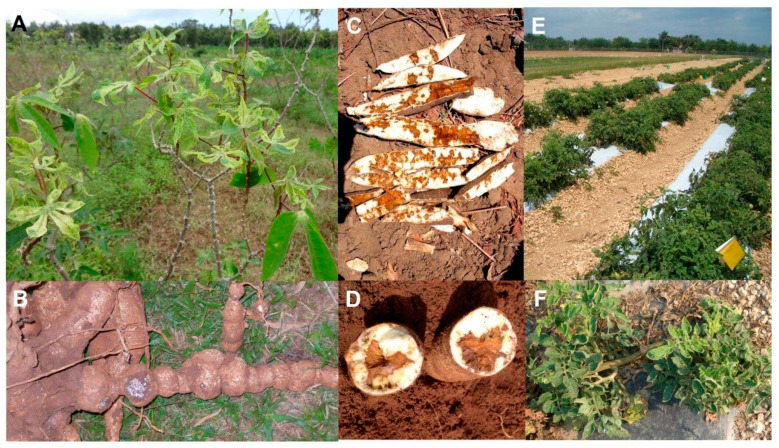
(**A**) Field of cassava devastated by cassava mosaic disease. Remaining upper leaves on diseased, mostly defoliated plants show symptoms consisting of severe mosaic and leaf deformation, image modified from [7]. (**B**) Roots of cassava showing marked constrictions caused by cassava brown streak disease (CBSD) (image credit @Natural Resource Institute/Maruthi Gowda). (**C**) Tuberous roots of cassava cut along their lengths showing dry necrotic rotting caused by CBSD (image credit @Natural Resource Institute/Maruthi Gowda). (**D**) Tuberous roots of cassava cut in cross section of showing dry necrotic rotting caused by CBSD (image credit @Natural Resource Institute/Maruthi Gowda). (**E**) Field of tomato devastated by tomato yellow leaf curl disease (TYLCD). All plants have symptoms of diminished leaf size, bunched growth, plant stunting, and lack of fruit formation, image modified from [5]. (**F**) Tomato plant showing severe symptoms of small, pale and upcurled leaves, bunched growth and plant stunting caused by TYLCD following early growth stage infection.

CMD was first found in 1984 in East Africa. By the 1940s, its presence had been reported in most SSA countries that grow this crop (Table 1). It now occurs in all SSA countries where cassava is grown, and, through trade in contaminated cassava cuttings, has spread to islands adjoining Africa [52,54,55,80]. Up until the early 1980s, attempts to manage CMD were restricted to places where its epidemics threatened rural livelihoods and caused food insecurity. These epidemics occurred in vulnerable cultivars. Unfortunately, such cultivars were generally the ones most preferred by smallholder farmers. This was due to the greater yields of higher quality tuberous roots they produced when harvested from healthy plantings [19,20,54,83]. After a virulent form of CMD that affected vulnerable cultivars very severely emerged in the late 1980s and infected cassava cuttings were planted widely, a highly destructive CMD epidemic arose in Uganda. It caused devastating losses in tuberous root production. Many rural inhabitants suffered an almost complete income loss, food shortages developed and famine-induced deaths occurred [19,20,23,102]. It then spread from Uganda to 10 other countries in East and Central Africa resulting in a disastrous CMD pandemic which caused enormous economic losses often accompanied by acute famine [19,20,23,52,55,83,103]. The foliage symptoms associated with CMD consist of severe leaf mosaic and deformation (Figure 1A), and plant stunting, sometimes resulting in plant death [104]. Up to 85% losses in tuberous root yields develop in CMD-affected plants of sensitive cultivars. However, some less widely grown cultivars are more tolerant, and so suffer smaller yield losses [20,105]. 

In 1983, the first CMD causal agent was described, African cassava mosaic virus (ACMV; genus *Begomovirus,* family, *Geminiviridae*). During the period 1983–2012, five further begomoviruses associated with CMD were found in SSA and one in Madagascar. All seven cassava begomoviruses were persistently transmitted by the polyphagous cryptic whitefly complex *Bemisia tabaci* [19,54,82,106]. Further, several recombinant strains derived from these begomoviruses were identified, and several alternative wild cassava begomovirus hosts belonging to the *Euphorbiaceae* or *Fabaceae* were reported in different parts of mainland SSA [54,80,107]. However, none of the seven begomoviruses causing CMD in SSA, or Madagascar, occur in cassava’s South American domestication centre [82]. Instead, these CMD causing begomoviruses all emerged in new encounter scenarios by spillover of indigenous begomoviruses spread by whitefly vectors from naturally-infected wild host plants into cassava after this crop was first introduced to different parts of this region. For example, ACMV, EACMV, South African cassava mosaic virus and cassava mosaic Madagascar virus probably emerged in West Africa, East Africa, South Africa and Madagascar, respectively. East Africa may be a major center of cassava begomovirus diversity as four cassava begomoviruses apparently emerged there. Whitefly vectors were responsible for spreading viruses from local infected alternative wild hosts to cassava resulting in cassava begomovirus emergence [54]. 

Following its invasion by indigenous begomoviruses, a combination of diverse factors was responsible for the development of CMD as a major threat to SSA cassava production. These included widespread planting of vulnerable cassava cultivars, widescale distribution of infected cassava planting material, recombination generating virulent new variants, synergistic interactions resulting from mixed cassava begomovirus infections, and frequent introductions of polyphagous whitefly vector types able to reach super-abundant numbers even above 1000 m above sea level [20,54,106]. What was responsible for the virulent form of CMD that caused the highly destructive CMD pandemic that started in Uganda in the late 1980s? This was caused by recombination between EACMV and ACMV resulting in the highly virulent recombinant called the EAMCV-Uganda variant (EACMV-UG). When co-infection occurred between ACMV and EACMV-UG, a synergistic interaction between the two viruses greatly increased virus titer causing very severe disease symptoms [82,102]. Cassava planting material carrying this mixed infection spread rapidly resulting in the disastrous East and Central African CMD pandemic [19,20,23,103]. Moreover, when a cassava mosaic virus is accompanied by a DNA satellite, infection with both may further magnify CMD-induced losses. This is because satellite presence can not only enhance CMD symptom severity but also overcome CMD resistance locus CMD2 enabling infection to occur in otherwise CMD-resistant cassava cultivars or land races that carry it [108].

In the south of the Indian subcontinent and Sri Lanka, CMD also causes major cassava disease epidemics. The principal cassava begomovirus responsible for the epidemics in central and southern India is Indian cassava mosaic virus, and in Sri Lanka it is Sri Lankan cassava mosaic virus (SLCMV). However, SLCMV is also found in southern India [54,80,82]. In addition, CMD caused by SCLMV is currently emerging as an important disease of cassava in Southeast Asia. It was found first in 2016 in Cambodia, and then spread to Vietnam, Thailand and Southernmost China [84]. 

## 5. Cassava Brown Streak Disease

CBSD was recorded first in 1936 infecting cassava crops in coastal Tanzania (Table 1). By 1950, it was found at altitudes below 1000 m in coastal East and southern Africa, and inland in Malawi and Uganda [20,57]. For several decades it was mostly ignored, but this changed in the 1990s when it re-emerged as a major factor causing epidemics that greatly diminished production of unblemished cassava tuberous roots and threatened food security. This occurred first in the East African Lake Victoria region, and next in most countries of East Africa, including at altitudes over 1000 m. By 2010, CBSD had spread widely and was causing a pandemic resulting in devastating losses in cassava production in East and Central Africa. Moreover, the likelihood of its further spread posed a serious risk to West African cassava crops [52,57,83,109]. CBSD causes root constriction (Figure 1B) and a brown-black, necrotic rot of cassava tuberous roots (Figure 1C,D). In addition, CBSD diminishes yields of tuberous roots by up to 70%. Its foliage symptoms consist of chlorotic blotching, mottle and veinal chlorosis of leaves, and brown stem streaking, symptom severity varying between cassava cultivars. These foliage symptoms are often too subtle for farmers to recognize and asymptomatic infection also occurs, so disease presence often goes unnoticed within the growing crop. This leads to infected cuttings being distributed for transplanting and farmers not knowing their cassava crop is affected until after its tuberous roots are harvested [20,52,57,83,110]. 

The two CBSD causal agents are cassava brown streak virus (CBSV; genus *Ipomovirus;* family, *Potyviridae*) and the closely related Ugandan cassava brown streak virus (UCBSV). The whitefly *B. tabaci* transmits both semi-persistently [57,80,111,112,113,114]. The foliage and root symptoms that UCBSV elicits differ from those CBSV causes. UCBSV causes circular chlorotic blotches between leaf veins without any veinal associations, whereas CBSV elicits more severe root necrosis, and feathery chlorosis alongside veins from which chlorotic blotches develop [57]. There is as yet no evidence of recombination between CBSV and UCBSV but potentially synergistic mixed infection occurs commonly with both of them and may elicit more severe symptoms. The only alternative hosts reported are the wild perennial tree cassava (*Manihot glaziovii*) and the non-cassava wild species *Zanha africana* and *Trichodesma zeylanicum* in which CBSV was detected, and the wild cassava species *Manihot carthaginensis* in which both viruses were found. Whether these species act as virus reservoirs for CBSV and UCBSV spread to cassava crops is unknown but seems plausible [57,80,115]. CBSV and UCBSV only occur in Africa, and are indigenous ipomoviruses from tree cassava or wild host species occurring within Africa. They emerged in new encounter scenarios within Coastal East and southern Africa, and in areas below 1000 m in altitude inland within East and Central Africa. This emergence was by spillover of the two indigenous ipomoviruses from naturally-infected wild plants to cassava after it was introduced, whitefly vectors being responsible for spreading them to cassava [57,80,112,116]. The likely reasons for the CBSD pandemic disease threatening food security in East and Central Africa were as follows: (i) inadvertent transportation of CBSD-infected cassava planting material to many new locations; (ii) distribution of vulnerable cultivars likely including CMD-resistant ones that turned out later to be CBSD-susceptible; and (iii) frequent introductions of polyphagous whitefly vectors capable of reaching superabundance at over 1000 m above sea level [20,52,54,57,116].

## 6. Tomato Yellow Leaf Curl Disease

The most important vegetable crop grown worldwide is tomato. It is important for human nutrition as it provides the human body with vitamins, minerals and plant compounds that bestow health benefits, including antioxidants. Ancestral cultivated tomato was originally confined to the central Andean region of South America (now in Peru and Ecuador), where it was first domesticated from wild tomato species. After spreading north in the Americas in pre-Columbian times, its domestication continued in Mexico. It was taken from there to Europe in the 16th century from where it was later distributed globally [117]. At least 136 virus diseases affect the tomato crop [118]. An example of a tomato virus disease pandemic that arose from virus spillover and is now endangering food security in developing countries worldwide is described below. 

Globally, the most economically significant tomato virus disease is tomato yellow leaf curl disease (TYLCD) (Table 1). It occurs in the world’s tropical and subtropical regions where its epidemics collectively cause a devastating pandemic, which is often the principal factor limiting tomato production. It was reported first in Israel in the 1930s and has severely damaged tomato crops in Middle Eastern countries since the 1960s. It remained restricted to Middle Eastern and eastern Mediterranean countries until the late 1980s. However, in the three decades that followed it spread west to the Western Mediterranean region, Caribbean islands, Central America, North America, and Venezuela in northern South America; south to West and East Africa, and to Reunion Island and Mauritius in the Indian Ocean; and east to the Arabian peninsula, the Indian subcontinent, Southeast Asia and East Asia; and then in 2006 to Oceania [80,91,92,93]. TYLCV symptoms in tomato foliage consist of leaf upward curling, yellowing and diminished size, flower abortion and plant stunting (Figure 1E,F). TYLCD epidemics cause dramatic losses due to the greatly decreased number of fruit formed. When early plant infection is widespread, the order of magnitude fruit yield loss can reach 100% causing total crop failure. Since tomato is often a major component of the diet of smallholder farmers in many developing countries, severe TYLCD outbreaks in their crops leads to hunger, indebtedness and farm abandonment [80,91,92,93]. 

The causal agent of TYLCD is tomato yellow leaf virus (TYLCV; genus *Begomovirus,* family, *Geminiviridae*) which is persistently transmitted by the whitefly *B. tabaci*. Tomato is its primary host, but it also naturally infects some alternative hosts sporadically, including common bean (*Phaseolus vulgaris*), the solanaceous ornamentals petunia (*Petunia hybrida*) and lisanthus (*Eustoma* spp.), and several wild tomato species [71,80]. TYLCV itself is subdivided into seven distinct strains but only the mild (Mld) and Israel (IL) strains have been dispersed widely outside the Middle East [92]. *B. tabaci* is a polyphagous supervector that exists as a species complex. Its cryptic species MEAM1 (= biotype B) and MED (= biotype Q) are its most efficient transmitters [68,92,119]. In the field, *B. tabaci* transmits TYLCV from infected to healthy plants both locally and, when viruliferous whitefly are blown over greater distances in wind currents. Some strain TYLCV-IL variants may be seed transmitted in tomato [120]. 

TYLCV is an indigenous virus to the Middle East. Somewhere in between the Jordan Valley eastwards to Iran, it emerged in a new encounter scenario at the interface between natural and managed vegetation. This emergence occurred by spillover from unidentified indigenous TYLCV-infected host sources into the introduced tomato crop. As mentioned above, tomato was domesticated in the Andean Region of South America. Factors favoring its emergence from infected indigenous plant hosts included efficient vector transmission by the cryptic species of the *B. tabaci* complex present and TYLCV’s ability to infect tomato crop plants readily [71,121]. Its widespread dissemination and establishment globally has been attributed to international trade in tomato seedlings unknowingly infected with TYLCV and infested with viruliferous *B. tabaci* MEAM1 or MED cryptic species. In addition, inadvertent international trade in TYLCV-infected tomato fruits and seedlings might also have been implicated [4,68,92,121,122]. 

Several other begomoviruses that infect tomato, and are relatives of TYLCV, cause TYLCD locally in some world regions. This includes tomato yellow leaf curl China virus, tomato leaf curl Malaysia virus; tomato yellow leaf curl Kanchanaburi virus, tomato yellow leaf curl Malaga virus, tomato yellow leaf curl New Delhi virus, tomato yellow leaf curl Sardinia virus, and tomato yellow leaf curl Thailand virus [53,68,80,122,123,124,125,126]. However, TYLCV is generally more invasive than these other tomato begomoviruses, which are mostly restricted to regions in which they are indigenous, so it tends to displace them [127]. 

## 7. Groundnut Rosette Disease

Grain legume crops such as peanut (=groundnut; *Arachis hypogaea*) are important for human nutrition and achieving sustainable food production. Their greater use would improve food security considerably not only by improving human and livestock health but also by improving soil fertility through fixing atmospheric nitrogen [128]. Currently, one of the major factors holding back their wider usage is lack of consistently in obtaining high yields regularly due to virus disease epidemics [129,130,131]. This applies to grain legumes grown in warmer climates such as peanut, common bean, cowpea (*Vigna unguiculata)*, mung bean (*Vigna radiata*), and soybean (*Glycine max*), and cool season grain legumes, such as chickpea (*Cicer arietinum*), faba bean (*Vicia faba*), field pea (*Pisum sativum*), lentil (*Lens culinaris*) and lupin (*Lupinus* spp.) [131,132,133]. An example of a major grain legume virus disease epidemic that arose by virus spillover, and now endangers food security in developing countries is described below. 

**Figure 2:**
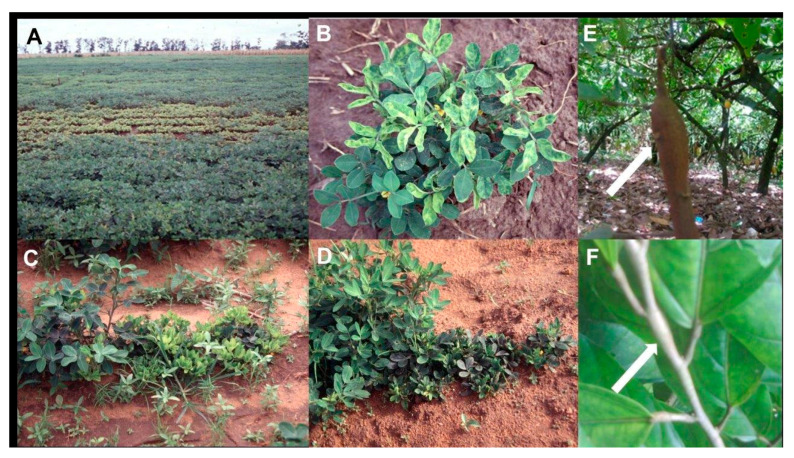
(**A**) Field of peanut with a large central area of chlorotic severely stunted plants caused by the chlorotic rosette syndrome of groundnut rosette disease (GRD) (image credit @Washington State University/Naidu Rayapati). (**B**) Peanut plant showing chlorotic (yellow) leaf mosaic symptoms caused by the chlorotic rosette syndrome of GRD (image credit @Washington State University/Naidu Rayapati). (**C**) Row of peanut plants showing bushiness and severe plant stunting caused by the chlorotic rosette syndrome of GRD (right and center), healthy plant on left (image credit @Washington state University/Naidu Rayapati). (**D**) Row of peanut plants showing bushiness and severe plant stunting caused by the green rosette syndrome of GRD (right), healthy plants on left (image credit @Washington state University/Naidu Rayapati). (**E**) Cacao tree showing swollen trunk symptom (pointed to by arrow) caused by cacao swollen shoot disease (CSSD) (image credit @International Institute of Tropical Agriculture/Lava Kumar). (**F**) Shoot of cacao tree showing characteristic swollen shoot symptom (pointed to by arrow) caused by CSSD (image credit @International Institute of Tropical Agriculture/Lava Kumar).

Peanut is an important crop that helps to ensure developing country food security. More than 6000 years ago, it was domesticated independently in several locations in South America. It was introduced to SSA in the 16th century [134]. The most important virus disease of peanut in SSA is groundnut rosette disease (GRD) [87,88]. It was first reported in Tanzania in 1907, and later elsewhere in SSA and its offshore islands, including Madagascar (Table 1). It causes a destructive disease in many countries of East, West, Central and southern SSA, and Madagascar [19,87,88,89]. Although generally sporadic in occurrence, GRD epidemics can be very damaging resulting in almost total peanut crop failure, and their unpredictability greatly hampers attempts to manage the disease. In semiarid tropical conditions, they cause yield losses of greater magnitude than any other peanut virus disease. GRD poses a very serious constraint to peanut production, and its epidemics often cripple the rural economy, causing smallholder farmers to abandon growing the crop [19,87,88,89]. GRD foliage symptoms consist of two main types, ‘chlorotic rosette’ (chlorotic yellow leaf mosaic and rosette; Figure 2A–C) and ‘green rosette’ (green leaf mosaic and rosette; Figure 2D). Chlorotic rosette occurs throughout SSA, but green rosette is less widely distributed. Both types of rosette syndromes cause young diseased plants to appear bushy, and become severely stunted. Diseased older plants only develop symptoms in some of their shoots or parts of their shoots. Yield losses are greatest in young plants, and can reach 100% resulting in complete crop failure when widespread infection occurs before flowering time [88].

GRD is elicited by a tripartite virus complex consisting of groundnut rosette virus (GRV; genus, *Umbravirus,* family, *Tombusviridae*), groundnut rosette assistor virus (GRAV; genus, *Luteovirus,* family, *Luteoviridae*) and satellite RNA (sGRV). *Aphis craccivora* (the cowpea aphid) transmits this tripartite virus complex persistently. It is not seed borne. Presence of GRAV is essential for transmission of GRV and sGRV to occur. Infection with GRAV and GRV but without sGRV fails to elicit any symptoms since sGRV is required for symptom expression [19,87,88,89]. The legume crops common bean, cowpea, soybean and mung bean, and two weed species, *Physalis peruviana* and *Cassia obtusa* are potential alternative hosts for the tripartite virus complex [90]. Carry-over of infection between growing seasons may occur in infected volunteer peanut plants or infected alternative host species. In addition to these infection sources, overlapping plantings of old infected peanut crops also act as reservoirs of the virus complex for spread to new crops within the growing season. *A. craccivora* vectors spread this complex to the peanut crop [19,87]. The GRAV, GRV and sGRV complex is indigenous to SSA. However, GRAV itself is also present on its own in the Indian subcontinent, Southeast Asia and Oceania [80]. The GRAV, GRV and sGRV complex emerged in SSA by spillover from infected wild vegetation spread by its aphid vector to the peanut crop after this crop was introduced [19,87,88,89]. Which factors favored development of the GRD epidemics in peanut crops in SSA? Thresh [19] referred to several cultural practices preferred by smallholder farmers that would have contributed to this. These included sowing peanut late in the growing season following the cereal (normally maize) harvest and using wide row spacing to save scarce seed supplies. *A. craccivora* vectors and virus reservoirs were most abundant at this stage of the growing season and wide row spacing attracted incoming aphids to land on peanut plants, both of which favored virus spread. For various reasons, GRD control recommendations to sow earlier using narrow row spacing proved too inconvenient to be adopted by the smallholder farmers.

## 8. Cacao Swollen Shoot Disease

Cacao (*Theobroma cacao*) is an evergreen, understory tree indigenous to the Amazonian rain forest in South America. It was introduced to West Africa in the second half of the 19th Century where it was mostly planted in lowland forest areas [1]. Its beans are very important to the global confectionery industry as both chocolate and cocoa powder are derived from the cocoa butter extract obtained from them. Cacao plantations therefore provide an important source of income for farmers in developing countries. An example of a cacao virus disease pandemic that arose by virus spillover is described below. 

CSSD was reported first in 1936 in Ghana, West Africa, but had been present for many years beforehand causing a widespread tree dieback syndrome (Table 1). Due to its dependence on cocoa as an export crop, by killing millions of trees, the CSSD pandemic caused enormous losses to Ghana’s economy, other West Africa countries, such as the Ivory Coast, Nigeria and Togo, experiencing similar losses [18,20]. Indeed, during the period 1946–1997, the Ghanaian eradication campaign against CSSD had cut down 193 million trees [18,20]. By 2020, CSSD has still not been contained effectively, despite this eradication program having being underway for >70 years and constituting the most costly such virus eradication program ever anywhere in the world [85,86]. CSSD’s symptoms in infected cacao trees include swelling of the trunk (Figure 2E) and at shoot nodes, internodes (Figure 2F) or tips and on roots, leaf chlorosis and vein banding, and tree dieback [85,86]. CSSD reduces cacao bean yields by 25% in the initial infection year, 50% in the second year and normally within 3–4 years then proceeds to kill cacao trees [135].

CSSD is caused by cacao swollen shoot virus (CSSV; Genus, *Badnavirus,* family, *Caulimoviridae*), which is transmitted semi-persistently by several mealybug species. Its most efficient mealybug vectors are *Planococcoides njalensis* and *P. citri*. Its alternative natural hosts are five indigenous West African tree species, *Adansonia digitata*, *Ceiba pentandra*, *Cola gigantean*, *C. chlamydanta*, and *Sterculia tragacantha*. Its mealybug vectors spread it from infected to healthy trees [85,136,137]. CSSV emerged at the managed and natural vegetation interface in West Africa by spillover from is indigenous tree hosts into cacao trees introduced from Amazonia. In eastern Ghana, this is thought to have involved the native forest understory tree *C. chlamydanta* as the virus source because this tree species is commonly CSSV-infected and colonized by mealybugs, grows in close proximity to cacao plantings and was infected by the same CSSV strain as that found in nearby cacao trees [1]. In other countries, it is unclear whether alternative indigenous CSSV host species other than *C. chlamydanta* growing near cacao plantings were involved in its emergence, as, where this issue was studied, spread by mealybug vectors possibly occurred to them from infected cacao instead of in the opposite direction [136]. 

Unfortunately, the initial West African CSSV epidemics were exacerbated by another factor as almost all the first large-sale plantings were a monoculture of cacao cv. Amelonado, which had come directly from Amazonia. Although ideal for producing high quality cacao beans and growing well in West African lowland forest areas, this cultivar proved very vulnerable to CSSV infection which rapidly kills it. Although more CSSV-tolerant cacao cultivars with resistance to mealybugs were introduced subsequently, in 2006 there were still large areas of cv. Amelonado plantings in West Africa likely to suffer damaging CSSV epidemics [20]. Thus, given the significance of cacao beans as the only source of the key ingredient for chocolate and its confections, cacao provides an example of a globally important crop being threatened by a highly damaging pandemic resulting from introduction of a new crop to another continent, and being aggravated by large-scale planting of a vulnerable cultivar.

## 9. Other Virus Diseases

Table 1 provides details of three other major virus disease epidemics arising from spillover of indigenous viruses from infected wild plants into introduced cereal crops of critical importance for global food security. These crops are (i) maize, which is not only the world’s most important staple food crop overall, but also the third most important in the developing world [77,138]; and (ii) Asian rice, which, conversely, is not only the developing world’s most important staple food crop, but also the third most important overall (see Section 3). Maize was domesticated in Mexico. Following the Spanish conquest of the Americas in the 15th century, it was dispersed from there to other continents reaching Europe and Africa in the 16th and 17th centuries, respectively [139]. 

Maize streak disease (MSD) and maize rough dwarf disease (MRDD) both arose by new encounters at the managed and natural vegetation interface (Table 1). This was by spillover of the indigenous viruses maize streak virus (MSV, genus *Mastrevirus,* family, *Geminiviridae*) and maize rough dwarf virus (MRDV; genus *Fijivirus,* family, *Reoviridae*) spread by their respective hopper vectors from infected *Digitaria* and other wild grass species. With MSV, its leafhopper vectors *Cicadulina mbila* and nine other *Cicadulina* species, and with MRDV, its planthopper vector *Laodelphax striatellus*. With MSV, this spillover occurred in southern Africa [97,98], but with MRDV in Southern Europe and the Middle East [1,20,94,95]. With MSV, the main trigger for its emergence was recombination between its virus strains resulting in virulent recombinant strain MSV-A, which adapted readily to its new host maize [98]. Another important factor contributing to the development of disastrous MSD epidemics was agricultural intensification, including widespread use of vulnerable short-season maize hybrids enabling two overlapping maize crops to be grown per year. Having two crops per year allowed its leafhopper vectors to spread MSV readily from one crop to the next [98]. With MRDV, its emergence by spillover from wild grasses to maize likely occurred well before the 1940s when it caused devastating epidemics in Italian maize crops sown with recently introduced high yielding American cultivars. These American maize cultivars were much more vulnerable than those grown previously. The same scenario unfolded in maize crops in Israel in the 1950s [1,20]. Currently, MRDD outbreaks caused by MRDV remain a major threat to maize production throughout the Mediterranean region [96]. 

Rice hoja blanca disease (RHBD) arose in northern South America from a new encounter scenario at the managed and natural vegetation interface. It involved spillover of the indigenous rice hoja blanca virus (RHBV, genus *Tenuivirus, family, Phenuiviridae*) spread by its vector planthopper *Sogatodes orizicola* from RHBV-infected plants of *Echinochloa colona* and other wild grasses to rice (Table 1) [78,99]. This spillover to rice likely occurred well before the 1930s when RHBD was recognized as the cause of major rice virus disease epidemics in Colombia. Within two decades, similar disastrous RHBD epidemics occurred in Venezuela, Panama, Costa Rica, Cuba and Florida, and within a further decade throughout subtropical and tropical regions of Americas. RHBVs rapid widespread dissemination was caused by long-distance flights of viruliferous *S. orizicola* leafhoppers. The devastating crop losses that occurred resulted from widespread use of highly RHBV- and *S. orizicola*-susceptible rice cultivars, and intensive cultural practices, such as continuous rice cropping, which favored RHBV spread [99]. 

Instead of becoming infected by indigenous virus spillover occurring directly from local virus-infected native plants, introduced crops can also become invaded indirectly by virus spread from infection reservoirs consisting of infected plants belonging to indigenous crops, crops introduced previously or introduced weeds [4]. For example, after papaya’s introduction from the Americas to Eurasia, the global papaya ringspot disease pandemic that papaya ringspot virus (PRSV, genus, *Potyvirus,* family, *Potyviridae*) elicited is considered to have started in the Indian subcontinent. Aphid vectors spread the virus from PRSV-infected cucurbit crop plants already growing there to papaya plants growing in nearby plantations of this introduced tree crop. PRSV’s host adaptation to papaya was attributed to a mutation that enabled cucurbit-adapted PRSV to infect it readily [140]. Such indirect virus spillover via an intermediate crop host seems more likely to occur with generalist than specialist viruses due to their broader host ranges [4].

There are many other examples of food security being impaired by major plant virus disease epidemics that arose after introduced crops domesticated in one continent were introduced to another where indigenous viruses they had never met before infected them. Such emerging virus disease epidemics often result from infection with viruses in the *Begomovirus*, *Orthotospovirus* and *Potyvirus* genera [1,2,4,7,19,20,25,71,125]. Moreover, as viruses belonging to these three genera are often generalists, with them it is normally unclear whether indigenous virus spillover occurred directly or indirectly into the introduced crop. An example of this is provided by groundnut bud necrosis virus (GBNV; genus *Orthotospovirus,* family, *Tospoviridae*), which is indigenous to the Indian subcontinent and has a wide natural host range. After introduction of the originally South American crop tomato (see Section 6) to the Indian subcontinent and Southeast Asia, GBNV infected it causing a disastrous major epidemic [141,142,143]. Whether its spillover into tomato was directly from GBNV infection reservoirs in indigenous native plants, or indirectly via such reservoirs already present in other crops or introduced weeds, is unknown. 

Begomovirus disease complexes have caused major epidemics in introduced and local vegetable crops growing in Southeast Asia [126]; the Indian subcontinent [53,144]; the Middle East and Mediterranean region [67,145]; SSA [146]; Northern South America [147,148,149,150]; and both Central America and Mexico [151]. The many indigenous begomoviruses that occur locally in tomato outside its domestication center, have infected it by indirect or direct spillover from local infection reservoirs. There is evidence some indigenous begomoviruses that infect tomato in Brazil have infected it by direct spillover from wild plant hosts [71]. However, although alternative non-crop hosts of some of these begomoviruses have been identified [53,71,126], in general, whether the original spillover to tomato events occurred directly from such infected wild hosts or indirectly via other already infected crop plants or introduced weeds remains to be determined. However, the critical role played by recombination and pseudo-recombination in begomovirus adaptation to tomato as a new host is well established, e.g., in Southeast Asia [126], the Indian subcontinent [53] and South America [71]. Expansion and intensification of tomato production, and the introduction of the more efficient *B. tabaci* MEAM1 whitefly vector were critical factors in subsequent tomato begomovirus epidemic development [71,122,126,147,152].

## 10. Management

Preventing initial spillover events that trigger virus emergence from occurring in a newly introduced crop might be possible initially if small-scale plots can be grown on farms where rigorous hygiene standards are maintained. The new crop would have to be grown in such a way as to avoid any possibility of an indigenous virus spreading into it from potentially virus-infected crop or wild plant alternative hosts, and would need regular inspections and virus testing to identify and destroy any potentially virus-infected plants. However, preventing initial spillover events in this way would be extremely difficult or impossible to achieve in practice once the scale of production increases and the crop is being grown widely in different regions, especially in developing countries.

Once a damaging emerging virus disease pandemic or epidemic initiated by spillover is underway in one region of the world, it is important to prevent, or failing that, minimize, further spread of the virus, or virus complex causing it. This requires measures designed to prevent it from entering, establishing or spreading to, and within, other, regions or continents. Strict biosecurity and plant health measures are needed to achieve this. Such measures include quarantine restrictions applied in the exporting country (pre-border), and at land borders, seaports and airports (border), along with virus eradication and containment programs (post border) [153]. 

Within regions where a virus disease epidemic is already underway, there is no ‘one-size-fits-all’ approach towards managing its spread within affected farms and fields. What is required to achieve this is devising integrated virus disease management strategies and tactics that suppress its spread most effectively [7,15,16,19,38,130]. These involve the deployment of appropriate combinations of phytosanitary, cultural, chemical and host resistance measures that target different components of the disease cycle and operate in different ways; biological control measures are sometimes included too, but most suited to protected cropping systems [7,15,16,19]. Such integrated strategies must be adjusted to take into account the scale and nature of the agricultural or horticultural production system involved which may vary from smallholder scale to very large-scale, and also according to climatic conditions, and local ecosystem and societal constraints [7,15,16,19]. Thresh [19,38] described what needs to be done to optimize the effectiveness of integrated disease management in tropical regions, including for most of the devastating virus disease pandemics and epidemics described in this review. Unfortunately, his guidance over adopting an integrated approach was often neglected in the past, especially in SSA. This was due to a tendency to focus on breeding crops for virus resistance and chemical control, whilst neglecting phytosanitary and cultural control measures (CSSD being a notable exception to this because of the widely adopted eradication (phytosanitary) campaign against it). Recently, there are signs this situation is changing, e.g., the widescale development of healthy cassava stock programs as a phytosanitary control measure against CMD and CBSD in SSA [82,83], and the inclusion of some phytosanitary and cultural control measures within the integrated disease management approaches Rojas et al. [119] recommended for geminivirus diseases.

Achieving greater success in managing virus disease pandemics or major epidemics in introduced crops after their initiation by indigenous virus spillover events, requires the strengthening of existing collaborative multidisciplinary research networks developed to address them. Where this is currently absent for a virus disease pandemic currently underway, the creation and fostering of a new collaborative network is warranted. Such multidisciplinary networks require collaboration between developed and developing country researchers, and the participation, amongst others, of specialist plant virologists, entomologists, modelers, agronomists, plant breeders, statisticians, and socioeconomics experts [7]. An example of a collaborative network addressing plant virus disease pandemics initiated by spillover events currently threatening food security in SSA is one tackling CMD and CSSD in cassava [83]. 

## 11. Conclusions

Improving the human food supply by introducing food crops domesticated in one continent to another continent, or to another part of the same continent, has exposed a major drawback to this endeavor. This drawback is the unforeseen development of damaging virus disease pandemics or major epidemics that arise by spillover of indigenous viruses into the introduced crops once they become established in their new surroundings. This review provides graphic examples of the enormous crop damage, gross yield and quality losses, and harm to the dependent human population that result. Most of the affected introduced crop examples described were domesticated in the Americas (maize, cassava, peanut, tomato, cacao) and distributed elsewhere in the world as part of the Columbian Exchange, but one of them originated in China (rice), being dispersed from there to other continents. Apart from cacao, these are all staple food crops vitally important for food security in developing countries. Moreover, apart from tomato, all are best suited to growing in regions with tropical or subtropical climates, most of which occur in food insecure parts of the developing world. Tomato not only grows well in such regions but also in warm temperate regions and under protected cropping in cool temperate regions.

Historical and up-to-date descriptions are provided of four examples of virus disease pandemics, and two examples of major epidemics, that arose from spillover scenarios involving new encounters between introduced crops and indigenous viruses spreading from infected natural vegetation. Five of these examples concern virus diseases of staple food crops that threaten food security in developing countries. These examples include those caused by CMD, CBSD, GRD, RYMD in SSA, and by TYLVD in all continents, apart from Antarctica. Because it devastates production of a valuable export crop, a sixth example caused by CSSD threatens livelihoods in West Africa. In addition, brief accounts are provided of other major virus disease epidemics arising from spillover scenarios involving new encounters in different parts of the world, namely MSD, MRDD, RHBD and several regional tomato orthotospovirus and begomovirus disease epidemics. For each pandemic or epidemic, the major factors driving its emergence initially, and its sudden increase in importance and geographical distribution subsequently, are explained. All these examples illustrate how spillover event initiated virus diseases epidemics that threaten food security in developing countries, vary greatly. This variation depends upon the characteristics of the causal virus(es), the crop affected, and the diversity of virus transmission modes, disease cycles, epidemiology, agro-ecological production systems and climatic factors involved. Tackling them successfully requires collaboration between policy makers, funding agencies, researchers and extension personnel on intercontinental scales. This scale of activity is needed to obtain a full understanding of each virus–vector–crop pathosystem and its epidemic drivers, and develop effective control measure approaches and extension strategies. Due to the urgent need to feed the growing global population, and address the increasing difficulties in controlling virus diseases of both staple and other food crops effectively as climate change progresses, the importance of the task ahead should not be underestimated.

## Figures and Tables

**Table 1:** Nine examples of virus disease pandemics or major epidemics of critical importance for global food security initiated by encounters between indigenous viruses and introduced crops.

Disease	Continents or Regions Currently Affected	Causal Agent(s)	Virus Genus	Vector(s)	Crop Diseased	Crop Origin	Impact	Virus(es) Origin(s)	Causes(s) of Emergence at Interface	Wild Spill over Hosts	Factors favouring Increased Importance/Distribution	Key Citations
Cassava brown streak disease	East, Central and southern Africa	Cassava brown streak and Uganda cassava brown streak viruses	*Ipomovirus*	Whitefly *(Bemisia tabaci*)	Cassava (*Manihot esculenta*)	Amazon rainforest	Widespread devastating yield losses	Coastal East and southern Africa; areas below 1000 m in altitude inland in East and Central Africa	Cassava introduction; spread by whitefly vectors	Wild tree cassava (*Manihot glaziovii*), unknown wild species	Growing vulnerable cultivars; trade in contaminated cassava cuttings; introductions of whitefly supervectors	[20,52,54,55,57,82,83]
Cassava mosaic disease	Sub-Saharan Africa and offshore islands, incl. Madagascar; (less affected so far: South India, Sri Lanka and Southeast Asia)	Cassava mosaic virus complex (seven viruses in sub-Saharan Africa/Madagascar. (Two further viruses in South India, Sri Lanka and Southeast Asia)	*Begomovirus*	Whitefly (*Bemisia tabaci*)	Cassava (*Manihot esculenta*)	Amazon rainforest	Widespread devastating yield losses; food shortages acute famine; deaths	Sub-Saharan Africa and Madagascar; East Africa a major center of diversity. (Indian subcontinent and Southeast Asia, separately)	Cassava introduction; spread by whitefly vectors	Several wild *Euphorbiaceae* and *Fabaceae* species	Growing vulnerable cultivars; trade in contaminated cassava cuttings; recombination generating virulent new variants; introductions of whitefly supervectors	[20,23,52,54,55,57,82,83,84]
Cacao swollen shoot disease	West Africa	Cacao swollen shoot virus	*Badnavirus*	Mealybugs (*Planococcoides njalensis* and *P. citri)*	Cacao (*Theobroma cacao*)	Amazon rainforest	Widespread devastating yield losses; infected trees soon killed. Most costly virus eradication program ever.	West Africa	Cacao introduction; spread by mealybug vectors	*Tree species Cola chlamydanta,* (*C. gigantean* *Adansonia digitata, Ceiba pentandra,* and *Sterculia tragacanth*a)	Large-sale planting of vulnerable cacao cv. Amelonado grown as a monoculture	[1,20,85,86]
Groundnut rosette disease	Sub-Saharan Africa and offshore islands, including Madagascar	Groundnut rosette virus, groundnut rosette assistor virus and virus satellite	*Umbravirus*, *Luteovrus*, virus satellite tri-partite complex	Aphid (*Aphis craccivora)*	Peanut (= groundnut), *Arachis hypogea*)	South America (several locations)	Devastating yield losses, crop failure; major deterrent to peanut cultivation	Sub-Saharan Africa	Peanut introduction; spread by aphid vectors	*Physalis peruviana and Cassia obtusa*	Cultural practices including late sowing, wide row spacing	[19,87,88,89,90]
Tomato yellow leaf curl disease	All continents except Antarctic	Tomato yellow leaf curl virus. (Several other begomoviruses also cause TYLCD, but have localised distributions—see Section 9)	*Begomovirus*	Whitefly *(Bemisia tabaci*)	Tomato (*Solanum esculentum*)	Andean region (Peru, Ecuador)	Widespread devastating yield losses, crop failure, hunger	Middle East and Iran	Tomato introduction; spread by efficient whitefly vectors	Several wild tomato species	International trade in tomato seedlings infected with TYLCV’s Mld and IL strains and carrying efficient whitefly vector MEAM1 and MED cryptic species; wind currents carrying viruliferous whitely vectors	[4,68,71,91,92,93]
Maize rough dwarf disease	Mediterranean region and Middle East	Maize rough dwarf virus	Fijivirus	Planthopper (*Laodelphax striatellus)*	Maize *(Zea mays)*	Mexico	Devastating yield losses in hybrid maize cultivars; major threat to maize crop	Mediterranean region and Middle East	Maize introduction; spread by planthopper vector	*Digitaria sanguinalis* and other wild grasses	Growing vulnerable hybrid maize cultivars	[1,20,94,95,96]
Maize streak disease	Sub-Saharan Africa	Maize streak mosaic virus	*Mastrevirus*	Leafhoppers (*Cicadulina mbila* and nine other *Cicadulina* species)	Maize *(Zea mays)*	Mexico	Widespread devastating yield losses, famine in some years	Southern Africa	Maize introduction; spread by leafhopper vectors; appearance of recombinant strain MSV-A	*Digitaria,* sp. and other wild grasses	Growing vulnerable short-season hybrid maize cultivars; agricultural intensification	[55,71,97,98]
Rice hoja blanca disease	South, Central and North America	Rice hoja blanca virus	*Tenuivirus*	Planthopper (*Sogatodes orizicola*)	Rice (*Oryza sativa*)	China	Devastating yield losses in some years	South America	Rice introduction; spread by its leafhopper vector	*Echinochloa colona* and other wild grasses	Intensified rice cropping; growing vulnerable rice cultivars; long-distance spread by viruliferous leafhopper vector	[78,99]
Rice yellow mottle disease	East, Central and West Africa, and Madagascar	Rice yellow mottle virus	*Sobemovirus*	Contact, beetles, mammals soil and water	Asian rice (*Oryza sativ*a) and African rice (*Oryza glaberrima)*	China	Widespread devastating yield losses; major deterrent to rice cultivation	East and West Africa	Spread by contact and vectors; intensification of rice production; Introduction of Asian rice	Wild rice: *Oryza barthii*, Orryza *longistaminata.* Wild grasses. *Echinochloa colona, Eragrostis atrovirens* and *Panicum repens*	Intensive irrigated rice production. Short-distance spread by beetle vectors, contaminated soil, irrigation water, machinery. Long-distance spread by trade in live rice seedlings, stubble and ratoons	[1,55,75,76,77,78,79]
